# Genetic and Environmental Factors Affecting TNF-α Responses in Relation to Sudden Infant Death Syndrome

**DOI:** 10.3389/fimmu.2015.00374

**Published:** 2015-07-27

**Authors:** Sophia M. Moscovis, Ann E. Gordon, Osama M. Al Madani, Maree Gleeson, Rodney J. Scott, Sharron T. Hall, Christine Burns, Caroline Blackwell

**Affiliations:** ^1^School of Biomedical Sciences, Faculty of Health and Medicine, University of Newcastle and Hunter Medical Research Institute, Newcastle, NSW, Australia; ^2^Hunter Medical Research Institute, New Lambton, NSW, Australia; ^3^Medical Microbiology, University of Edinburgh, Edinburgh, UK; ^4^Genetics, Hunter Area Pathology Service, John Hunter Hospital, New Lambton, NSW, Australia; ^5^Immunology, Hunter Area Pathology Service, John Hunter Hospital, New Lambton, NSW, Australia

**Keywords:** sudden infant death syndrome, TNF-α, ethnicity, cigarette smoke

## Abstract

Dysregulation of the inflammatory responses has been suggested to contribute to the events leading to sudden infant deaths. Our objectives were (1) to analyze a single nucleotide polymorphism (SNP) associated with high levels of tumor necrosis factor-α (TNF-α) responses, *TNF* G-308A, in sudden infant death syndrome (SIDS) infants, SIDS and control parents, and ethnic groups with different incidences of SIDS; (2) the effects of two risk factors for SIDS, cigarette smoke and virus infection, on TNF-α responses; and (3) to assess effects of genotype, cigarette smoke, and gender on TNF-α responses to bacterial toxins identified in SIDS infants. *TNF* G-308A genotypes were determined by real-time polymerase chain reaction for SIDS infants from Australia, Germany, and Hungary; parents of SIDS infants and their controls; and populations with high (Aboriginal Australian), medium (Caucasian), and low (Bangladeshi) SIDS incidences. Leukocytes from Caucasian donors were stimulated *in vitro* with endotoxin or toxic shock syndrome toxin-1 (TSST-1). TNF-α responses were measured by L929 bioassay (IU/ml) and assessed in relation to genotype, smoking status, and gender. There was a significantly higher proportion of the minor allele AA genotype among Australian SIDS infants (6/24, 24%) compared to 3/62 (4.8%) controls (*p* = 0.03). There were no significant differences in TNF-α responses by *TNF* G-308A genotypes when assessed in relation to smoking status or gender. Given the rarity of the *TNF* G-308A A allele in Caucasian populations, the finding that 24% of the Australian SIDS infants tested had this genotype requires further investigation and cautious interpretation. Although non-smokers with the AA genotype had higher TNFα responses to both TSST-1 and endotoxin, there were too few subjects with this rare allele to obtain statistically valid results. No effects of genotype, smoking, or gender were observed for TNF-α responses to these toxins.

## Introduction

Tumor necrosis factor-α (TNF-α) is a key pro-inflammatory cytokine which could play a role in several of the mechanisms proposed to explain sudden infant death syndrome (SIDS). In relation to the anaphylaxis hypothesis, TNF-α is stored in mast cell granules and is released on antigen stimulation. TNF-α also induces fever and affects respiration (1) and can affect myocardial function (2, 3). One of its single nucleotide polymorphisms (SNPs), *TNF* G-308A, has been associated with severe responses to infection. Cytokine gene polymorphisms can significantly affect the level of these substances produced in response to infection (4). *In vitro* studies found that the A allele was associated with increased responses (5) and individuals homozygous for the A allele had higher levels of circulating TNF-α (6). The molecular basis for the A allele being a more powerful transcriptional activator is not clear; however, the region −323 to −285 has been found to bind nuclear factors differently compared with the corresponding G allele (7).

Reports on the *TNF* G-308A SNP indicate the AA genotype carried an increased risk of death from cerebral malaria (8), septic shock (9), and death from meningococcal sepsis (10). Two studies examined SNPs for TNF-α among Scandinavian SIDS infants and found no significant association (11, 12). Because of the variability of reports on findings for a variety of SNPs among SIDS infants in different populations, we examined the *TNF* G-308A genotypes among material from our collection of samples from Germany, Hungary, and Australia. Because of the variation in the incidence of SIDS among different ethnic groups, we also included comparison groups from populations with low (South Asian), moderate (Caucasian), or high (Indigenous Australian) incidences of SIDS.

Although gene polymorphisms are important determinants of the cytokine responses, we have reported that three major risk factors associated with SIDS – gender, exposure to cigarette smoke, and virus infections – can significantly influence these responses (13–15). In this study, we used model systems to assess interactions between these risk factors and TNF-α responses elicited by components of bacterial species identified in SIDS infants, lipopolysaccharide (LPS) of Gram-negative species (16, 17), and toxic shock syndrome toxin-1 (TSST-1) of *Staphylococcus aureus* (18).

The objectives of the study were (1) to analyze the distribution of *TNF* G-308A alleles among SIDS infants, SIDS parents, an unrelated adult comparison group, and ethnic groups with different incidences of SIDS; (2) to assess effects of virus infection and cigarette smoke on TNF-α responses; and (3) to assess the effect of gender, *TNF* G-308A, and cigarette smoke on responses to bacterial antigens (LPS and TSST-1) identified in SIDS infants.

## Subjects and Methods

Approval for the study was obtained from the Lothian Health Ethics Committee (UK), Hunter Area Research Ethics Committee (Australia) and the University of Newcastle Human Research Ethics Committee (Australia).

### Assessment of *TNF* G-308A SNP

Buccal epithelial cells were collected from Caucasian parents of SIDS infants from Britain (*n* = 34) and Australia (*n* = 60) and a comparison group with no family history of sudden infant deaths (Britain *n* = 56, Australia *n* = 62). Paraffin-fixed samples of tissue from SIDS infants were obtained from Australia (*n* = 25), Hungary (*n* = 18), and Germany (*n* = 46). Stored frozen blood samples from Aboriginal Australians (*n* = 117) and buccal epithelial cells from Bangladeshi donors (*n* = 32) were used as sources of DNA for comparisons among ethnic groups. The methods for extraction of DNA from the samples have been described previously (13).

*TNF* G-308A (rs1800629) genotype was determined by a commercial allelic discrimination polymerase chain reaction (PCR) assay (Assay ID: C___7514879_10) (PE Applied Biosystems). Primers and probes were provided in a 20× assay mix, sequences and concentrations of which are unknown.

Each PCR reaction contained 10 ng of sample DNA, 1× Assay mix, and 1× TaqMan Universal PCR Master Mix (PE Applied Biosystems) made up to a final volume of 5 μl with sterilized MilliQ water. PCR was performed using the ABI PRISM 7900HT sequence detection system (PE Applied Biosystems) at the following thermal cycling conditions: 50°C for 2 min; 95°C for 10 min; 92°C for 15 s; and 60°C for 1 min, for 40 cycles.

Data were analyzed using the statistical software package Statistics/Data Analysis™ (STATA) Version 8.0 (Stata Corporation, College Station, USA). The Chi-square (χ^2^) test or Fisher’s Exact test, if appropriate, was used to assess the distribution of *TNF* G-308A in SIDS infants, parents of SIDS infants, and between ethnic groups. Genotype distribution from the Hardy–Weinberg equilibrium (HWE) was assessed using the χ^2^ test.

### Assessment of the effects of interferon-γ (IFN-γ) and cigarette smoke extract (CSE) on TNF-α responses elicited by endotoxin from THP-1 cells

Tumor necrosis factor-α responses of the THP-1 human monocytic cell line were measured as previously described in the model system to assess the effects of surrogates for infection (IFN-γ) and exposure to CSE on cells with a uniform genetic background (19).

### Assessment of the effects of IFN-γ and CSE on TNF-α responses from human peripheral blood monocytic cells

Collection, isolation, and storage of human peripheral blood monocytic cells (PBMC) have been described previously. Buffy coats from 14 male and 14 female donors, aged 20–55 years, were purchased from the Australian red cross blood service (ARCBS) (Sydney, Australia). Ethical permission was obtained from University of Newcastle Human Research Ethics Committee (H-229–0606) and ARCBS Ethics Committee (07-11NSW-07) for the purchase and use of human buffy coats for the purposes of the study. PBMC were collected from each donor for *in vitro* cytokine stimulation assays and plasma was collected for the assessment of cotinine for evidence of exposure to cigarette smoke, a confounding variable for altered cytokine responses. *TNF* G-308A genotype was determined as described above. Donors with detectable levels of cotinine were excluded from the analysis. Only ARCBS donor samples that were cleared for infectious agents were received. Leukocytes were collected and stored as described previously (15).

In our initial studies, TNF-α responses were examined with blood samples (10–20 ml) collected from British parents of SIDS infants (*n* = 34) and a comparison group, individuals unrelated to the families and who had no family history of SIDS (*n* = 59). DNA was extracted from the leukocytes and screened for the *TNF* G-308A alleles as described above. The leukocytes were assessed for inflammatory responses to TSST-1 and LPS as described previously. All samples were coded and tested without knowing the sex or smoking status of the donors. Leukocytes (1 × 10^6^ cells ml^−1^) were stimulated *in vitro* with 0.01 μg ml^−1^ or 1 μg ml^−1^ *Escherichia coli* LPS or 0.5 μg ml^−1^ TSST-1 (Sigma, Poole, Dorset, UK) for 24 h. Cell culture conditions have been described previously (13).

### Quantitative assessment of TNF-α

Production of biologically active TNF-α was assessed by bioassay with L929 cells as described previously. The results were expressed as international units derived from the standard curves obtained using a recombinant human TNF-α standard.

### Statistical methods

Student’s *t*-test was used on log_10_ -transformed data to assess differences in TNF-α responses for the various experimental conditions tested. The significance level for all tests was set at *p* < 0.05.

## Results

The distributions of genotypes among the different groups assessed are summarized in Table 1. Distributions were in HWE for each of the groups assessed.

**Table 1:** ***TNF* G-308A allele frequency distribution in the study populations**.

Ethnicity	Group		Allele frequency (%)			Sample size (*n*)	*p* value (*p*)
			GG	GA	AA	
British	SIDS	Parents	65	35	0	34	0.89
British	Control	Parents	66	32	2	56	
Australian	SIDS	Parents	57	40	3	60	0.22
Australian	Control	Parents	69	26	5	62	
Australian	SIDS	Infants	48	28	24	25	0.03^a^
Hungarian	SIDS	Infants	61	39	0	18	0.05^b^ 0.09^c^
German	SIDS	Infants	85	15	0	46	<0.01^d^
Combined	SIDS	Infants	70	24	7	89	0.41^e^
Bangladeshi	Control		94	6	0	32	0.01^f^
Aboriginal Australian	Control		99	1	0	117	<0.01^g^ 0.12^h^
Caucasian	Control		68	29	3	118	

*^a^Australian SIDS infants vs. Australian control parents*.

*^b^Hungarian SIDS infants vs. German SIDS infants*.

*^c^Hungarian SIDS infants vs. Australian SIDS infants*.

*^d^German SIDS infants vs. Australian SIDS infants*.

*^e^Combined SIDS infants vs. Caucasian control*.

*^f^Bangladeshi control vs. Caucasian control*.

*^g^Aboriginal Australian control vs. Caucasian control*.

*^h^Aboriginal Australian control vs. Bangladeshi control*.

### Distribution of *TNF* G-308A among SIDS infants

The majority of each SIDS group possessed the GG genotype; however, Australian SIDS infants had a significant increase in proportion with the AA genotype (6/25, 24%) compared to the Hungarian (0/18, 0%) (*p* = 0.09) and German (0/46, 0%) (*p* < 0.01) SIDS infants.

The distribution of allele frequencies for the Australian control population differed significantly from that observed for Australian SIDS infants (*p* = 0.02). Only 3/62 (5%) of controls had the AA genotype compared with 24% of SIDS infants. No significant differences were detected between the distribution of *TNF* G-308A genotype for the combined SIDS infant group compared to the Caucasian controls (*p* = 0.41).

### Assessment of *TNF* G-308A among parents of SIDS infants

The distribution of *TNF* G-308A genotype among parents of SIDS infants was not significantly different compared with their respective control populations. Parents of SIDS infants recruited from Australia showed an increased proportion of individuals with the GA genotype compared to their matched Australian controls but this was not significant (*p* = 0.22). Parents of SIDS infants recruited from Britain had a genotype distribution similar to their control population; the majority of individuals had the GA genotype (*p* = 0.89).

### Distribution of *TNF* G-308A in different ethnic groups

The distribution of *TNF* G-308A varied significantly among individuals from different ethnic groups. The majority of both British and Australian control populations possessed the GG genotype with fewer than 5% of individuals possessing the AA genotype.

As there were no differences between the allele frequencies for British and Australian control populations, these data were combined for further comparison with the Bangladeshi and Aboriginal Australian populations. The distribution of allele frequencies differed significantly between the Caucasian group and both Bangladeshis (*p* = 0.01) and Aboriginal Australians (*p* < 0.01). More than 90% of the Bangladeshi and Aboriginal Australian populations possessed the GG genotype, and 0% had the AA genotype.

### Effects of INF-γ and CSE on TNF-α responses of THP-1 cells to LPS

**Figure 1:**
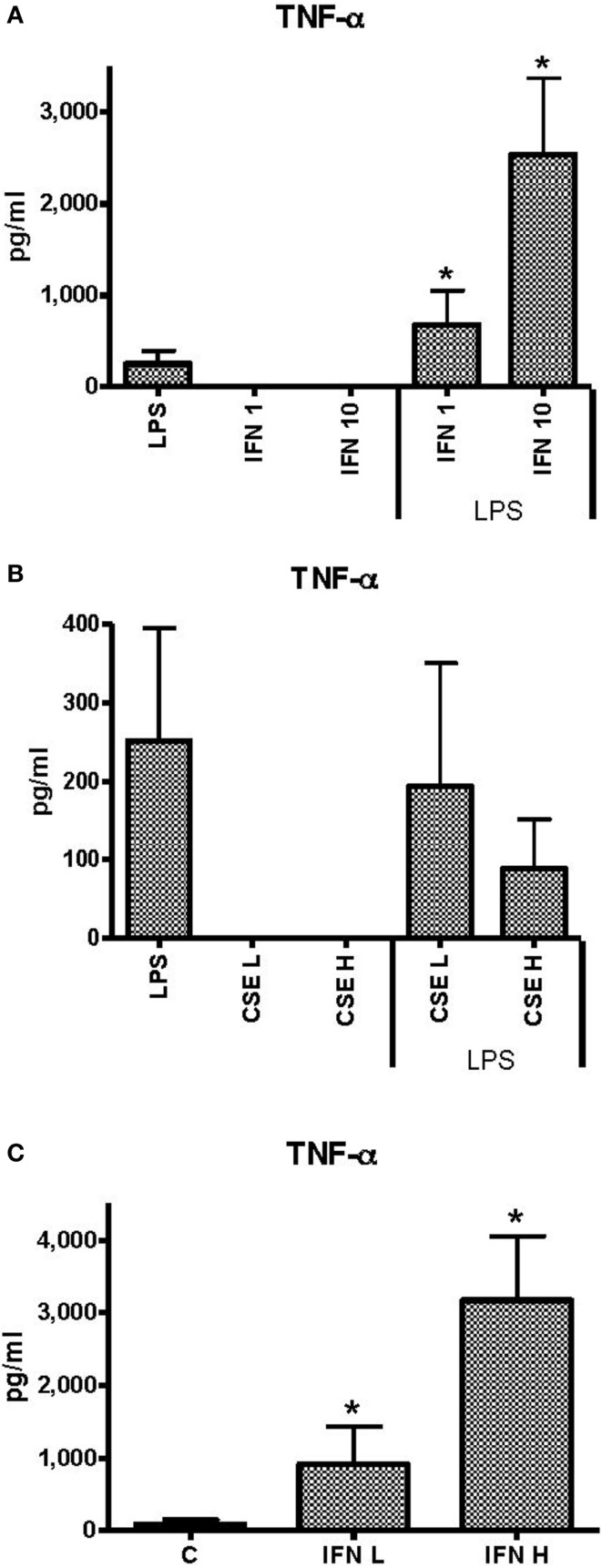
**Effects of IFN-γ and CSE on TNF-α responses of THP-1 cells to LPS (50 ng ml^−1^)**. **(A)** TNF-α responses of THP-1 cells to LPS (50 ng ml^−1^) and IFN-γ at 1 ng ml^−1^ (IFN 1) or 10 ng ml^−1^ (IFN 10) (*p < 0.05). **(B)** TNF-α responses of THP-1 cells to LPS (50 ng ml^−1^) and CSE at low level (CSE L = 0.0001 cig ml^−1^) and high level (CSE H = 0.001 cig ml^−1^). **(C)** TNF-α responses of THP-1 cell to LPS (50 ng ml^−1^) and CSE (0.001 cig ml^−1^) and IFN-γ at low level (IFN L = 1 ng ml^−1^) and high level (IFN H = 10 ng ml^−1^) (*p < 0.05).

Unstimulated THP-1 cells had no measurable TNF-α in response to INF-γ at 1 ng ml^−1^ (low) or 10 ng ml^−1^ (high) concentrations. Priming of THP-1 cells with INF-γ resulted in dose-dependent enhancement of TNF-α responses to LPS (50 ng ml^−1^) (Figure 1A). Neither low (L) (0.0001 cigarette per milliliter) nor high (H) (0.001 cigarette per milliliter) concentrations of CSE elicited measurable TNF-α. There was a dose-dependent but non-significant reduction in TNF-α responses to LPS in the presence of low dose of CSE (Figure 1B). In the presence of the high dose of CSE and either high or low concentrations of IFN-γ, TNF-α responses were significantly enhanced compared to stimulation with LPS alone (Figure 1C). The IFN-γ-enhanced TNF-α responses were not reduced by the CSE exposure.

### Effects of INF-γ and CSE on TNF-α responses of human PBMC to LPS

**Figure 2:**
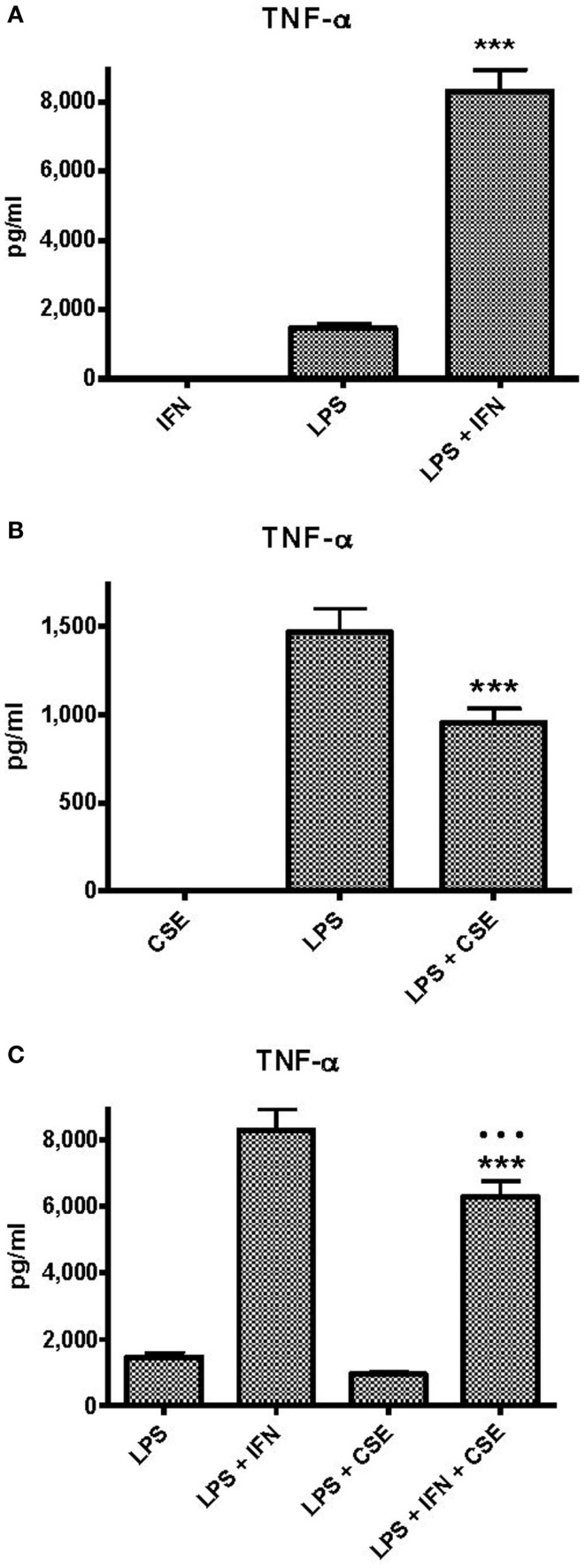
**Effects of IFN-γ and CSE on TNF-α responses of PBMC to LPS (50 ng ml^−1^)**. **(A)** Effect of IFN-γ (10 ng ml^−1^) pre-treatment on LPS stimulation of TNF-α from PBMC (*n* = 28) (*** =*p* < 0.0001). **(B)** The effect of CSE (0.001 cig ml^−1^) pre-treatment on LPS (50 ng ml^−1^) stimulation of TNF-α from PBMC (*n* = 28) (*** =*p* < 0.0001). **(C)** The effect of CSE (0.001 cig ml^−1^) and IFN-γ (10 ng ml^−1^) pre-treatment on LPS stimulation of TNF-α from PBMC (*n* = 28) (*** =*p* < 0.0001).

Peripheral blood monocytic cells from 28 blood donors who had no evidence of cigarette smoke exposure or infection were used for assessment of the effects of CSE and INF-γ on TNF-α responses to LPS in relation to genotype. Baseline levels of TNF-α responses elicited by the individual components and combinations of components were approximately twice those elicited from THP-1 cells (Figure 2A). INF-γ priming significantly enhanced TNF-α responses (*p* < 0.0001). As observed with THP-1 cells, CSE did not elicit TNF-α from PBMC. In contrast to the results obtained with THP-1 cells, the high dose of CSE significantly reduced TNF-α responses to LPS (*p* < 0.0001) (Figure 2B). The presence of both INF-γ and CSE resulted in enhanced TNF-α responses compared with LPS alone; however, the response was lower if pre-treated with CSE and INF-γ than if pre-treated treated with INF-γ alone (*p* < 0.0001). (Figure 2C).

When assessed by gender of the donor, there were no significant differences between: TNF-α responses to LPS, INF-γ, or CSE; or combinations of LPS with CSE or LPS with INF-γ. There was a significant difference in TNF-α responses of cells from female donors (6510 pg ml^−1^) compared with responses from cells of male donors (4621 pg ml^−1^) if both INF-γ and CSE were used to pre-treat cells prior to exposure to endotoxin (*p* = 0.014). There were no significant differences in TNF-α responses associated with genotypes GG (*n* = 15), GA (*n* = 10) or AA (*n* = 3) under any of the conditions tested. For donors with the GG genotype, there were higher responses to LPS from cells of female donors (*n* = 9) (1674 pg ml^−1^) than from cells of male donors (*n* = 6) (989 pg ml^−1^) (*p* = 0.03). While the responses from cells of females with the GA genotype (*n* = 2) were approximately twice that of those from cells of male donors (*n* = 8), the differences were not significant (*p* > 0.05). There were only three donors with the AA genotype and all were female.

### Effect of smoking, gender, and *TNF* G-308A on TNF-α responses to LPS or TSST-1

This experiment utilized cells from the UK donors. There were no significant differences in the median TNF-α response to TSST-1 or LPS associated with different genotypes of *TNF* G-308A for smokers (*n* = 38) or non-smokers (*n* = 60). For the GG and GA genotypes, the geometric mean TNF-α responses were below 10 IU ml^−1^. For the three donors with the AA genotype, there was one smoker and two non-smokers. The responses of the smoker donor with the AA genotype were similar to those of the GG and GA donors (<10 IU ml^−1^). The response of the two donors who were not smokers was nearly 20 IU ml^−1^ for responses to TSST-1 and over 50 IU ml^−1^ for responses to the higher dose of LPS. There were not enough donors with the AA genotype for statistical analyses.

## Discussion

In this study, we found an association with the *TNF* G-308A genotype AA in Australian SIDS infants. Given the rarity of the *TNF* G-308A A allele in the Caucasian population, it was an unusual observation that 24% of the Australian SIDS infants tested in this study were homozygous. The number of infants assessed in this study was small; therefore, data should be interpreted with caution. In the Norwegian study, 3% of SIDS infants, 5% of borderline SIDS, and 3% of infants who died of infection had the AA genotype; while none of the controls had this SNP genotype (11).

While the distribution of *TNF* G-308A genotype in the Indigenous Australian population differed significantly from that in the Caucasian population, the groups with high (Indigenous Australian) or low (UK Bangladeshi) incidences of SIDS had similar genetic profiles.

If the finding of a significant increase in the AA genotype associated with high TNF-α reflects a risk factor for SIDS, the findings need to be assessed in relation to the various hypotheses proposed to explain the physiological events leading to SIDS.

Long QT interval is reported to be an important risk factor for SIDS based on a prospective study of a large group of infants (20). The resident myocardial macrophages and mast cells and cardiomyocytes synthesize TNF-α. Arrhythmia has been reported as a side effect of treatment of patients with metastatic cancer with TNF-α, IL-2, and IFN-γ (21, 22). Negative ionotropic and arrhythmogenic effects were observed in myocytes cultured in IL-1, IL-2, IL-3, and TNF-α (23).

Dysregulation of glucose metabolism was proposed as one mechanism triggering the physiological events leading to SIDS (24). Acute hypoglycemia was associated with deranged cytokine levels. Hypoglycemia induced in rats with TNF-α without changes in the insulin levels was ameliorated by corticosteroid therapy (25). Hypoglycemia in an elderly patient with non-Hodgkin’s lymphoma was associated with normal insulin and insulin-like hormone levels but high TNF-α levels. Glucose homeostasis was normalized after lowering TNF-α by cytoreductive therapy (26).

The A allele has been associated with increased production of TNF-α (27, 28). The cytokine response, however, is multi-redundant and pleiotropic; therefore, inactivation or over production of a particular cytokine might not have an effect on its own. When combined with other imbalances in the cytokine cascade, real physiological differences could be observed. It is, therefore, important to interpret cytokine and cytokine SNP data with caution and to consider the effects of other genetic, developmental, and environmental influences on these responses.

Despite finding interactions with smoking and cytokine gene polymorphisms in our previous studies (13), this study found no significant effects of gender, active smoking, or *TNF* G-308A genotype on TNF-α responses to TSST-1 or LPS in the different populations. Although the responses of the non-smokers with the AA genotype were higher than those observed for the other genotypes, there were too few donors with this genotype for statistical analyses. These studies need to be repeated with more samples from individuals with the rare genotype.

In the experiments with cells from healthy Australian blood donors which were controlled for exposure to cigarette smoke and infection, females generally had higher TNF-α responses than males (15). When assessed by gender, females with the AA genotype had the lowest responses to the different conditions tested. This is in contrast to the results with cells from UK donors in which the rare AA genotype had the highest TNF-α responses to either TSST-1 or LPS. There are several factors that could contribute to the differences. In the study with Australian donors, TNF-α was assessed by Bio-Rad 6-plex assays and the Luminex 200 analyzer; this method detects total TNF-α. TNF-α responses from cells of the UK donors were tested for cytotoxicity for the L929 cell line which measures the biologically active TNF-α. The numbers of Australian donors tested were also smaller than those in the UK study. The UK samples were not assessed for level of exposure to cigarette smoke or concurrent viral infections which we have demonstrated can significantly alter cytokine responses including TNF-α. For the donors in the UK study, exposure to cigarette smoke was assessed only by self-reported smoking.

In conclusion, the *TNF* G-308A genotype results indicated that for the Australian population, infants with the AA genotype might be at increased risk of SIDS. The study needs to be expanded and investigations need to include thorough investigation for microorganisms and their toxins as well as investigation of the genetics and cytokine responses. While the effects of genotype on TNF-α responses remain unclear in the models tested, the effects of INF-γ are unequivocal for both THP-1 cells and PBMC. The results indicate that virus infection might enhance TNF-α responses to minor bacterial infection. While exposure to cigarette smoke is a risk factor for SIDS, it does not appear to enhance TNF-α responses to LPS. For the PBMC studies, CSE reduced significantly the enhanced responses of IFN-γ primed cells to LPS.

## Author Contributions

Each of the authors made substantial contributions to the conception, design, analyses, and interpretations of the work. They assisted in preparing the article, critically assessed the final version and agree to be accountable for the accuracy and integrity of the work.

## Conflict of Interest Statement

The authors declare that the research was conducted in the absence of any commercial or financial relationships that could be construed as a potential conflict of interest.
